# Supplementary material for: Isolation and Characterization of a New Endophytic Actinobacterium Streptomyces californicus Strain ADR1 as a Promising Source of Anti-Bacterial, Anti-Biofilm and Antioxidant Metabolites
Source: Microorganisms. 2020 Jun 19;8(6):929. doi: 10.3390/microorganisms8060929 (PMC7355773; doi:10.3390/microorganisms8060929)
Supplement: Supplementary file 1 [file microorganisms-08-00929-s001.pdf]

Figure S1.

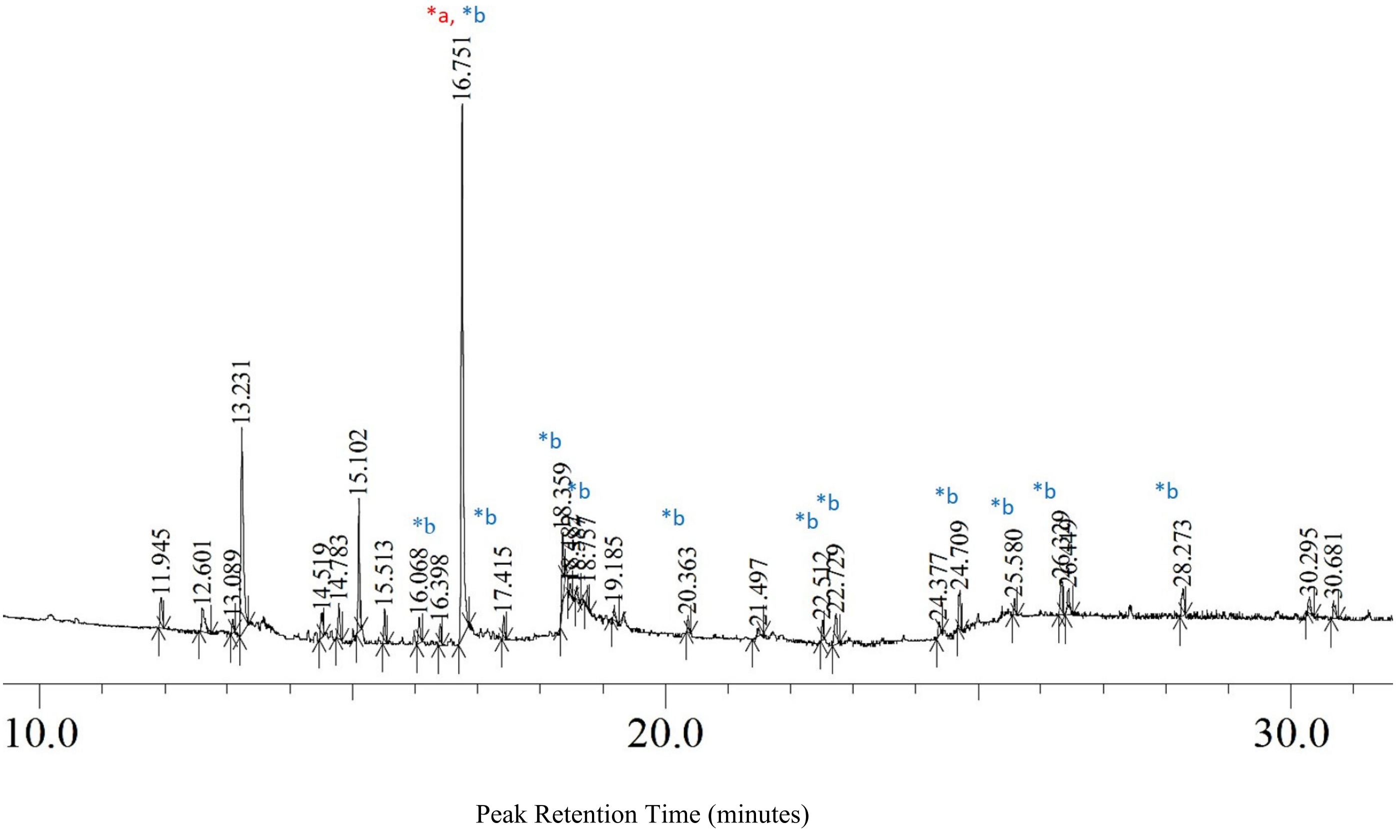

Figure S1. GC-MS Chromatogram of ADR1 metabolites. The specific peaks have been identified by the RT. No peaks were observed after 31 minutes.

* a. Solvent specific peaks, * b. Production medium specific peaks

Table S1. Compounds detected in the ADR1 metabolites extract by GC-MS analysis using NIST & Wiley library as reference

S. No.	RT	SI	Reference Compounds from Library
1.	11.945	88	Methanoazulen-9-ol, decahydro-2,2,4,8-tetramethyl-stereoisomer
2.	12.601	78	2-[(Trimethylsilyl)oxy] propan-1-ol
3.	13.089	96	Naphtho [2,3-g]-1,6,2,5-dioxasilaborocin
4.	13.231	84	2-[(trimethylsilyl)oxy]-4-methoxyacetophenone
5.	14.519	90	5-z-methyl-2-z-hydroxycarbonyl-5-e-ethenyl-4-z-propen-2-ylcyclohexanone
6.	14.783	75	trimethylsilyl ester of tetradecanoic acid
7.	15.102	92	Diisobutyl phthalate
8.	15.513	76	n-Pentadecanoic acid
9.	16.068	88	2-pyridineethanamine, n-methyl-n-[2-(4-pyridinyl) ethyl]
10.	18.483	72	Methoxyacetic acid, 2-ethylhexyl ester
11.	18.483	73	3,3-Dimethyl-2-pentanol
12.	18.587	73	4-Phenylbutan-2-ol, trimethylsilyl ether
13.	18.680	65	Cyclohexylundecyl ester of Oxalic acid
14.	19.185	71	4-Ethyl-2-octanol
15.	21.497	72	Methyl 2-butynoate
16.	24.377	71	4-(4'-methylphenyl)-4-pentenal
17.	26.329	79	6,7-epoxy-3,7,11-trimethyldodeca-1,3,10-triene
18.	26.449	79	2-(2-methylidenecyclopropyl) propan-2-yl (E)-but-2-enoate
19.	30.295	NA	No hits found
20.	30.681	63	Trimethylsilyl ester of 2,6-dihydroxybenzoic acid
